# SwimOne. New Device for Determining Instantaneous Power and Propulsive Forces in Swimming

**DOI:** 10.3390/s20247169

**Published:** 2020-12-14

**Authors:** Francisco Hermosilla, Lis Corral-Gómez, José M. González-Ravé, Daniel Juárez Santos-García, David Rodríguez-Rosa, Sergio Juárez-Pérez, Fernando J. Castillo-Garcia

**Affiliations:** 1 Sport Training Laboratory, Faculty of Sports Sciences, University of Castilla-La Mancha, 45071 Toledo, Spain; fhermosilla@nebrija.es (F.H.); josemaria.gonzalez@uclm.es (J.M.G.-R.); daniel.juarez@uclm.es (D.J.S.-G.); 2 Facultad de Lenguas y Educación, Universidad Nebrija, 28015 Madrid, Spain; 3 School of Industrial and Aerospace Engineering, University of Castilla-La Mancha, 45071 Toledo, Spain; lis.corral@uclm.es (L.C.-G.); David.RRosa@uclm.es (D.R.-R.); Sergio.Juarez@uclm.es (S.J.-P.)

**Keywords:** training, swimming, force, power, resistance

## Abstract

The propulsive forces and instantaneous power that are generated by a swimmer have a great influence on the swimming performance. This works presents a new device, called SwimOne, for measuring propulsive force and estimating the instantaneous power of the swimmer. In addition, the detailed prototype is able to exert a customizable opposition force to the swimmer for training purpose. The conceptual idea is presented by describing the differential equation of the swimmer and the protocol for a factible estimation of the instantaneous power. The variables that are to be measured and estimated are identified and, consequently, the sensor and actuator systems can be selected. The high-level and detailed designs of the prototype are presented together with the protocol that is carried out in order to validate the sensor and actuation systems. The device is able to monitor the variables of interest of the swimmer together with the propulsive force and instant power. Finally, some experiments are carried out providing the results of several participants swimming in crawl, backstroke, butterfly, and breaststroke styles in the presence of different opposition force. The preliminary results show that SwimOne is valid for measuring instantaneous force and power with different loads in swimming.

## 1. Introduction

The estimation of propulsive forces is still an open problem for determining swimming performance. The propulsive forces that are applied by a swimmer affect swimming performance and are, therefore, among the key variables of interest in swimming research [1,2,3]. Nowadays, the direct measurement of propulsive forces during swimming has not been solved and, in practice, researchers have attempted to measure them during tethered swimming [4,5].

Custom-made devices are often used for this purpose and the participants swim while wearing a belt or harness that is attached to force-recording equipment through a non-extendable cable. With no forward swimming movement during such tests and, hence, no active drag, it is assumed that the forces recorded represent the propulsive forces of the swimmers. Some of the most reliable methods for assessing propulsive forces in swimming in the last years have been developed under tethered and semi-tethered scenearios [4,6,7].

In these sense, the power rack [8] is the most popular tethered and semi-tethered swimming device. A power rack is a system of pulleys and weights tethered to a swimmer by a waist belt attached to a cable, which, in turn, is attached to a load. When the swimmer performs a trial, the cable lifts the weight. The protocol for the specific strength swimming test was standardized in [9]. However, power rack has two major drawbacks: first of all, the use of multiple pulleys that impedes an objective measurement of force production, and the absence of photocells that are attached to the technology for measuring the time that is required for the swimmer to produce a determined distance. In addition, other technologies have been implemented with the use of load cell instead of weights, as a better and feasible way for measuring the force production.

In addition, tethered swimming is not completely similar to free swimming. In free swimming conditions, when the arm is in the propulsive phase of the stroke cycle, the upper arm and shoulder are moving forwards relative to the water and, therefore, encounter resistive drag [10]. It leads to kinematic changes that could change hands trajectories or accelerations comparing with real swimming [11]. It should be also considered that when the force goes to zero, the cable becomes slack, and the tensioning of the cable could result in a stress wave that will cause a pike in the force measurement [4].

Despite some interesting findings in the tethered swimming literature, there is still a scarcity of data about new devices which measuring objectively propulsive forces. The device that is presented here is also able to estimate the drag forces, providing reliable data about specific resistance of the swimmer and the maximum and average force performed during the swimming.

Thus, the aim of the current study is to present and validate the novel device for determining instantaneous propulsive force and power. In addition, the device can be used as training system toward improving the performance of the swimmers, evaluating the force that is exerted by a swimmer with different resistive forces [12,13].

## 2. Materials and Methods

### 2.1. Conceptual Design

**Figure 1:**
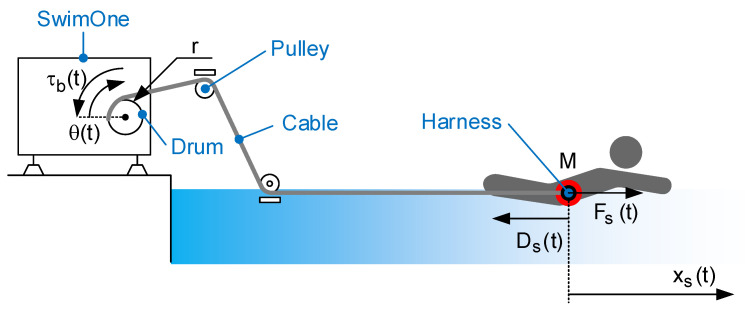
SwimOne: Conceptual design.

SwimOne is a portable device that must be installed at the edge of the pool. SwimOne is mechanically connected to the swimmer be means of a cable. A harness is used for this purpose. During the advance of the swimmer, the cable rolls out from a drum. SwimOne estimates the swimmer displacement, $x_{s}\left(t\right)$, and the instantaneous force, $F_{s}\left(t\right)$ in order to determine the instantaneous power during the exercise. The swimmer displacement is estimated by measuring the drum rotation angle, θ(t):

(1) $$x_{s}\left(t\right)=r\cdot \theta \left(t\right)$$

with *r* being the effective radius of the drum. The instantaneous force can be obtained by registering the cable tension $T_{c}\left(t\right)$. Figure 1 represents the conceptual design that has yielded SwimOne.

In addition, SwimOne is able to exert an opposite force, $F_{b}\left(t\right)$, to the swimmer by exerting an opposite torque in the drum axis, $\tau_{b}\left(t\right)=F_{b}\left(t\right)\cdot r$. Therefore, the dynamics equation of the swimmer movement can be described by:

(2) $$M\cdot {\ddot{x}}_{s}\left(t\right)=F_{s}\left(t\right)-\frac{\tau_{b}\left(t\right)}{r}-D_{s}\left(t\right)$$

where *M* is the swimmer mass and $D_{s}\left(t\right)$ is the water resistance or drag that is defined as ’the force on an object moving in a fluid due to the rate of change in momentum of the fluid influenced by the object moving through the fluid’ [14,15].

According to [16], drag, $D_{s}$, can be estimated by the following expression:

(3) $$D_{s}\left(t\right)=K\cdot {\dot{x}}_{s}^{2}\left(t\right)$$

with *K* being a constant that depends of the swimming style: Front Crawl, K=30.0 kg/m; Backstroke, K=26.9 kg/m; Butterfly, K=28.5 kg/m; and, Breaststroke, K=37.5 kg/m. Note that Equation (3) is an approximation, because it uses the instantaneous velocity of the swimmer, but a constant value of *K*, which only depends on the stroke.

The equilibrium of forces at any transmission pulley can be used to determine the cable tension, $T_{c}\left(t\right)$, which can be expressed as:

(4) $$T_{c}\left(t\right)=M\cdot \ddot{x_{s}}\left(t\right)-F_{s}\left(t\right)+D_{s}\left(t\right)$$

which allows for obtaining the swimmer propulsive force, as:

(5) $$F_{s}\left(t\right)=M\cdot {\ddot{x}}_{s}\left(t\right)+D_{s}\left(t\right)-T_{c}\left(t\right)$$

Finally, once that swimmer displacement and instantaneous force are estimated, instantaneous power, $P_{s}\left(t\right)$, can be determined by:

(6) $$P_{s}\left(t\right)=F_{s}\left(t\right)\cdot {\dot{x}}_{s}\left(t\right)$$

In summary, the procedure that is proposed here to estimate the instantaneous power of a swimmer is:

1. To measure the drum angle, θ(t), and:
  (a) to estimate the swimmer displacement, $x_{s}\left(t\right)=\theta \cdot r$.
  (b) To obtain swimmer velocity and acceleration, $v_{s}={\dot{x}}_{s}$ and $a_{s}={\ddot{x}}_{s}$.
  (c) To determine drag term $D_{s}\left(t\right)=K\cdot {\dot{x}}_{s}^{2}$.
2. To measure cable tension, $T_{c}\left(t\right)$, and to estimate propulsive force by means of $F_{s}\left(t\right)=M\cdot {\ddot{x}}_{s}\left(t\right)+D_{s}\left(t\right)-T_{c}\left(t\right)$.
3. To obtain instantaneous power by computing $P_{s}\left(t\right)=F_{s}\left(t\right)\cdot {\dot{x}}_{s}\left(t\right)$

The following sections detail the particular requirements for SwimOne device and the procedure and instruments to measure drum rotation angle θ(t) and cable tension $T_{c}\left(t\right)$ and exert an opposition torque, $\tau_{b}\left(t\right)$.

### 2.2. Requirements for Designing

The design requirements are:

1. It shall offer configurable swim resistance. The average force exerted by a swimmer in tethered trials are approximately 100−150 N, depending on the stroke [17,18,19,20]. In this way, the resistance force that the device is able to exert has been designed to be into the range [35–153] N.
2. It shall be able to monitor force, displacement, velocity, and instant acceleration to know the swimmer performance.

By knowing the temporal evolution of a set of variables of interest of swimmers, several strategies for improving their performance can be evaluated while using the SwimOne device.

### 2.3. High Level Design

**Figure 2:**
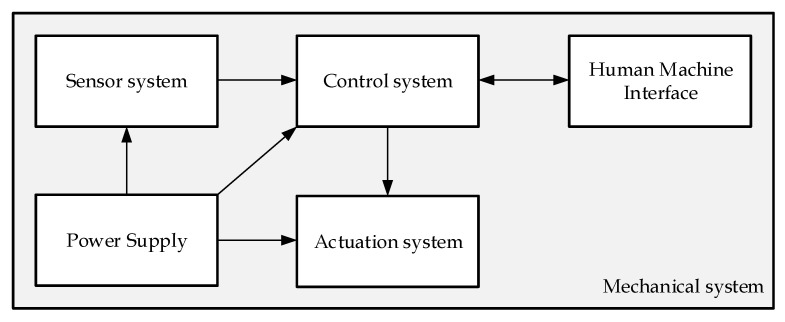
SwimOne: High Level Design.

Figure 2 represents the High Level Desing of SwimOne.

The device is formed by:

- Mechanical system. The mechanical system consists of the structure, cover case, and other mechanical elementary elements. This system includes a drum to roll in and out a cable that can be attached to the swimmer.
- Sensor system. This system should measure all of the required variables for estimating the instantaneous power of swimming (see Section 2.2). In this sense, a rotatory encoder has been selected to measure the angular position of the drum, θ(t). The instantaneous displacement swimmer can, therefore, be estimated by $x_{s}\left(t\right)=r\cdot \theta \left(t\right)$, with *r* being the effective radius of the drum, which depends of the quantity of cable rolled in the drum. The instant velocity, $v_{s}\left(t\right)={\dot{x}}_{s}\left(t\right)$, and acceleration, $a_{s}\left(t\right)={\ddot{x}}_{s}\left(t\right)$, can be estimated by differentiation of the displacement.
  The rotatory encoder is a digital sensor and, consequently, the differentiation of $x_{s}\left(t\right)$ includes non-desirable noise. A mobile mean filter has been implemented in order to remove the the noise in the estimation of $v_{s}\left(t\right)$ and $a_{s}\left(t\right)$ (see Section 3.2.1).
  This system also includes a torquemeter in order to estimate the instantaneous cable tension, $T_{c}\left(t\right)$.
- Actuation system. This system includes a DC motor to automated roll in or out the cable and a magnetic breaker to exert the configurable resistant force.
- Control system. The control system is a PLC (Programmable Logic Controller), which obtains the information of the sensors and command the behaviour of the actuation system. The sample time for registering (inputs) or commanding (outputs) the signal has been set to 20 ms.
- Human–Machine Interface. This system allows for the final user to configure the experiments and obtain the experimental data.
- Power Supply. It provides the required power supply to the control system, sensors, and actuators.

Section 3 details the calibration and validation procedure of both actuator and sensor systems.

### 2.4. Detailed Design

#### 2.4.1. Overall Design

**Figure 3:**
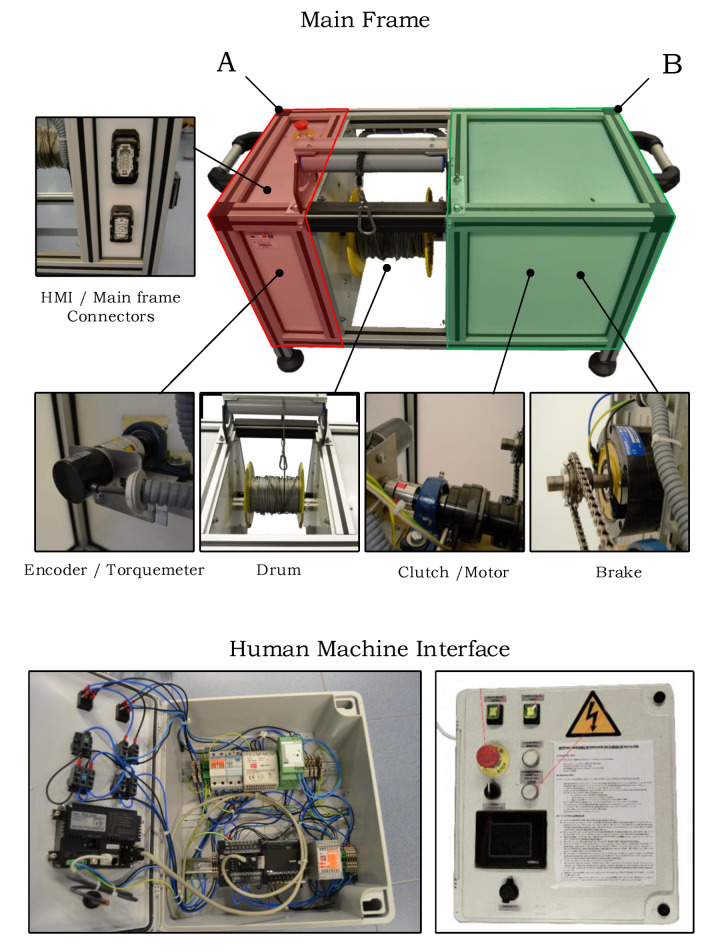
Final aspect of SwimOne.

SwimOne is an electrical–mechanical device that applies a resistive force to the swimmer and records the variable of interest for determining the instantaneous force and power of the swimmer. Figure 3 shows the final aspect of the device.

SwimOne has a drum that allows for rolling out and in the cable that is connected to the swimmer. The main frame consists of two watertight sections with IP68. Section A includes the encoder and torquemeter, since Section B includes the brake, the motor, and a clutch. The clutch is used to uncouple the motor during the experiments. Finally, the human machine interface includes a safety switch and all of the buttons and selectors to command the device. It also includes a touch panel that allows for configuring experiments and a USB-port to extract the data.

The main frame is connected to the control panel by means of two six-meter cables with Harting type connectors that ensure tightness.

To carry out a test, the user must set the force value that the swimmer will have to overcome, and then the brake applies the required torque in order to provide that force. When the test concludes, the software outputs a table with the values of force, velocity, and elapsed time.

#### 2.4.2. Actuator System

One of the main purposes of SwimOne is to exert a customizable force of opposition to the swimmer. For this purpose, a the electromagnetic brake, MEROBEL FAT650RR model, has been selected.

This electromagnetic brake is able to exert an opposition torque up to 65 Nm, demanding only 1 A of rated current. The electromagnetic brake has been mounted by a chain-based transmission with no reduction. The equivalent opposition force is estimated by means of:

(7) $$F_{b}=\frac{\tau_{b}}{r(\theta )}$$

with $F_{b}$ being the opposition force and $\tau_{b}$ the opposition torque.

#### 2.4.3. Sensor System

As mentioned in High Level Design Section, an encoder is used in order to measure the angular position of the drum and, consequently, to estimate the swimmer instant displacement. An incremental quadrature encoder OMROM E6B2-CWZ6C with a resolution of 1000 Pulse/Revolution has been selected.

Assuming that the initial pose of the swimmer, $x_{s}=x_{s0}=0$ m, is related to the initial angular position of the encoder, $\theta =\theta_{0}$ rad, the swimmer displacement is equivalent to the quantity of cable that is rolled out of the drum, i.e., $x_{s}\left(t\right)=r\left(\theta \right)\cdot \theta$. The effective radius of the drum, *r*, depends of the quantity of cable that is rolled out and, therefore, of the rotation angle of the drum.

Once the swimmer displacement is obtained, the instant velocity and acceleration can be estimated computing the first and second order derivative of the displacement. A mobile mean filter (centered and non-causal with a 11 samples) is applied to estimate both velocity and acceleration.

Finally, for estimating the instant force that is exerted by the swimmer, a torquemeter has been installed in the rotary axis of the drum.

This torquemeter outputs a ±5 V that is related to the torque range of the device (0–50 Nm).

## 3. Validation and Results

### 3.1. Effective Radius of the Drum

The linear displacement, $x_{s}\left(t\right)$, is estimated by measuring the drum rotation angle, θ(t), as shown in Figure 1. As a conventional drum has been used in SwimOne device, the effective radius of the drum decreases when the cable rolls out. This radius variation affects to the estimation of the swimmer displacement, $x_{s}\left(t\right)$, and, consequently, to the propulsive force $F_{s}\left(t\right)$. This section states the procedure to obtain $x_{s}\left(t\right)$ by the measurement of θ(t).

**Figure 4:**
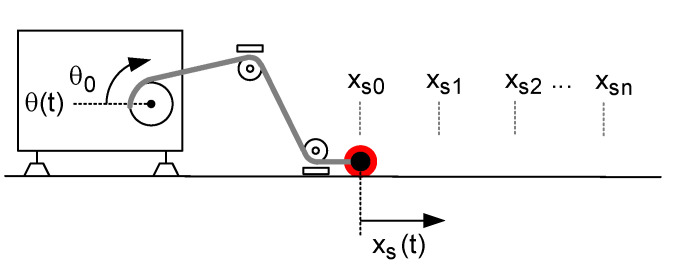
Scenario for obtaining θ(x).

From an initial pose, θ=0 rad and $x_{s}=0$ m, while using a tape measure, the tip of the cable has been placed in known positions each 0.50 m up to 20 m (as shown in Figure 4). For each known position ($x_{s1}$, $x_{s2}$, ...$x_{sn}$), the encoder measurement has been registered, obtaining the equivalent rotation angle measures ($\theta_{1}$, $\theta_{2}$, ..., $\theta_{n}$). Because the way that cable rolls in or out affects the $\theta_{i}$ values, this procedure has been repeated five times and for each value of $x_{s}$ the mean of the correspondent five values of θ has been obtained. Using the least squares method, the relationship between $x_{s}$ and θ can be determined. The resulting 4^th^ order regression is:

(8) $$x_{s}=8.5733\cdot 10^{-8}\cdot \theta^{3}-1.07214\cdot 10^{-4}\cdot \theta^{2}+7.5900\cdot 10^{-2}\cdot \theta$$

**Figure 5:**
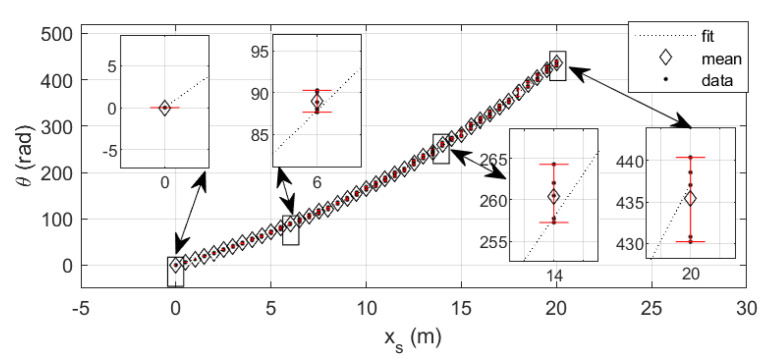
Experimental results for obtaining $x_{s}\left(\theta \right)$.

Figure 5 represents the obtained results, including the experimental data, the mean value of each set of θ values, and the regression. The results dispersion is larger when $x_{s}$ increases cause the cable rolls in the drum in different way in each attempt, producing a natural integral error, as shown in Figure 5.

**Figure 6:**
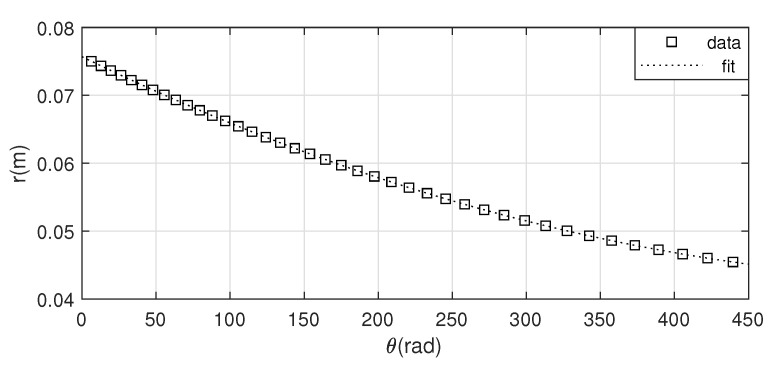
Experimental results for obtaining r(θ).

Once that the relationship between $x_{s}$ and θ is obtained, for each given angular position of the drum, the effective radius can be easily determined as $r=\frac{x_{s}}{\theta }$. Figure 6 represents the relationship between *r* and θ, which has also been approximated by the least square method, and it yields:

(9) $$r=8.486\cdot 10^{-8}\cdot \theta^{2}-1.061\cdot 10^{-4}\cdot \theta +7.570\cdot 10^{-2}$$

In summary, Equations (8) and (9) allow for obtaining the swimmer displacement, $x_{s}$, and the effective radius of the drum, *r*, by measuring the angular position of the drum axis, θ by means of the rotary encoder.

### 3.2. Sensor System Validation

#### 3.2.1. Displacement, Velocity and Acceleration

The device has been validated attaching the harness to a controlled movement mobile robot in order to validate the estimation procedure of instant displacement, velocity, and acceleration of the swimmer: NI Robotics Starter Kit. The encoder measurement is registered during the movement of the robot. The displacement, $x_{s}$, can be computed by Equation (8). A sample *i* of velocity, $v_{s}\left(i\right)$, and acceleration, $a_{s}\left(i\right)$, can, therefore, be estimated by discrete differentiation:

(10) $$\begin{array}{c} v_{s}\left(i\right)=\frac{x_{s}\left(i\right)-x_{s}\left(i-1\right)}{t(i)-t(i-1)} \end{array}$$

(11) $$\begin{array}{c} a_{s}\left(i\right)=\frac{v_{s}\left(i\right)-v_{s}\left(i-1\right)}{t(i)-t(i-1)} \end{array}$$

As the encoder is a digital sensor and the data obtained are used offline, a non-casual filter can be designed for improving the velocity and acceleration estimation. For avoiding delay in the filtered signal, a centered mobile mean filter has been applied for both velocity and acceleration. The size of the filter windows has been experimentally set to 11 samples, stating a compromise between the quality of the filter signal and computing time:

(12) $$\begin{array}{c} v_{s}^{f}\left(i\right)=\sum_{k=i-5}^{i+5}v_{s}\left(k\right) \end{array}$$

(13) $$\begin{array}{c} a_{s}^{f}\left(i\right)=\sum_{k=i-5}^{i+5}a_{s}\left(k\right) \end{array}$$

**Figure 7:**
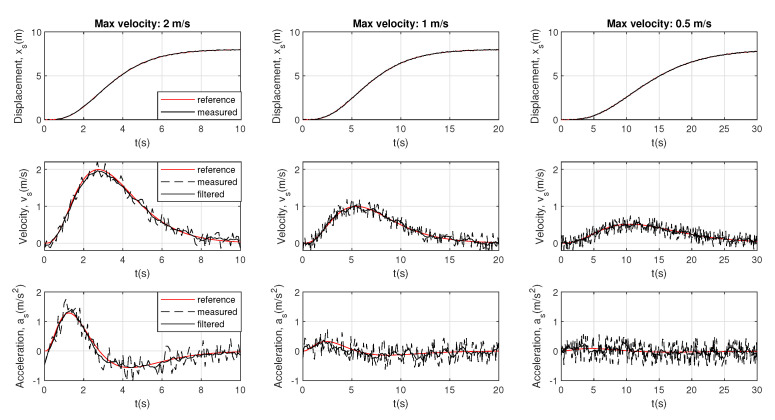
Results of displacement, velocity, and acceleration.

For validation purpose, a set of 20 dynamic experiments has been carried out from a maximum velocity of 0.1 to 2 m/s. Figure 7 illustrates three of these dynamic experiments (at 0.5, 1, and 2 m/s) comparing the reference of displacement, velocity, and acceleration, provided by the mobile robot that is attached to the harness, and the estimated ones.

**Figure 8:**
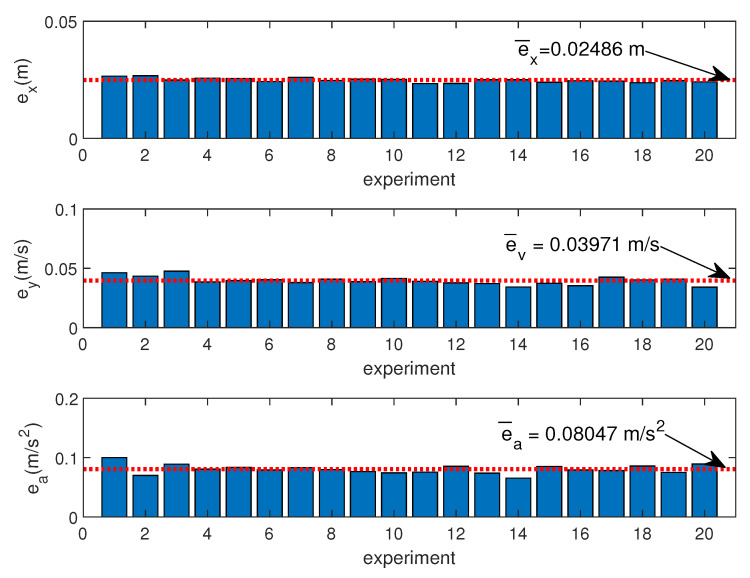
Displacement, velocity, and acceleration errors for different experiments.

The committed error in the displacement, velocity, and acceleration can be computed by the mean error, as:

(14) $$\begin{array}{c} e_{x}=\left(\sum_{k=1}^{n}\left|x_{s}^{*}-x_{s}\right|\right)/n \end{array}$$

(15) $$\begin{array}{c} e_{v}=\left(\sum_{k=1}^{n}\left|v_{s}^{*}-v_{s}\right|\right)/n \end{array}$$

(16) $$\begin{array}{c} e_{a}=\left(\sum_{k=1}^{n}\left|a_{s}^{*}-a_{s}\right|\right)/n \end{array}$$

where $x_{s}^{*}$, $v_{s}^{*}$, and $a_{s}^{*}$ are the reference of displacement, velocity, and acceleration, *n* the number of samples during each experiment, and $e_{x}$, $e_{v}$, and $e_{a}$ the mean error of the displacement, velocity, and acceleration, respectively. Figure 8 represents the $e_{x}$, $e_{y}$, and $e_{a}$ errors and their respective means for the 20 experiments. Note that the errors are almost constant for the 20 experiments (maximum velocity profile from 0.1 to 2 m/s), so the dynamics of the movements have no influence on the quality of $x_{s}$, $v_{s}$, and $a_{s}$ estimations.

#### 3.2.2. Cable Tension

The following procedure has been carried out in order to validate whether cable tension is properly measured by torquemeter described in Section 2.4.3. Firstly, the calibration curve voltage/torque is experimentally determined. For this purpose, the torquemeter is directly coupled to a pulley with a known radius, $r_{p}$, and connected to a set of known mass (m∈[1,50] Kg) in the vertical position by means of a cable. The reference torque, $\tau_{d}$ to be measured, can, therefore, be determined as:

(17) $$\tau_{d}=m\cdot g\cdot r_{p}$$

being *g* the gravity acceleration.

**Figure 9:**
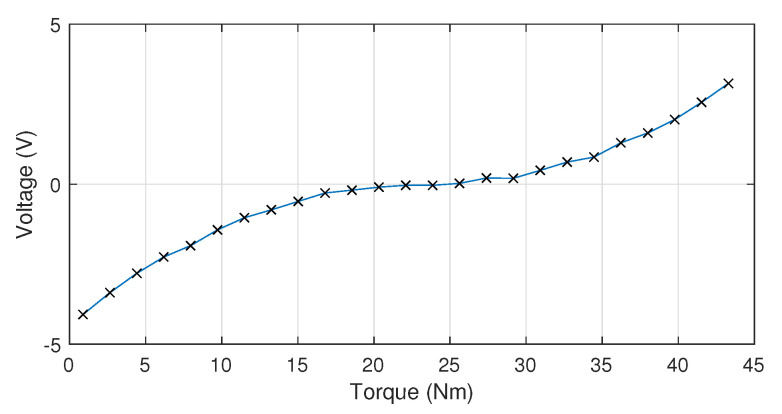
Torquemetercalibration curve: Voltage (V)/Torque (Nm).

Figure 9 represents the obtained calibration curve of the torquemeter, which relates the measured torque with the voltage that is acquired by the control system. The experimental results are very similar to the ones that were provided by the torquemeter supplying company.

Once that the relationship between reference voltage, *V*, and output torque, τ, has been determined, the drum is mechanically fixed at different angular positions, $\theta_{i}$, and for each angular position:

1. The effective radius of the pulley, *r*, is determined by (9).
2. A set of known mass (m∈[1,50] Kg) is connected to the drum in vertical position.
3. The cable tension is estimated by means of $T_{c}=\frac{\tau_{b}}{r}$ and compared with the reference tension that should be $T_{c}^{*}=m\cdot g$.

**Figure 10:**
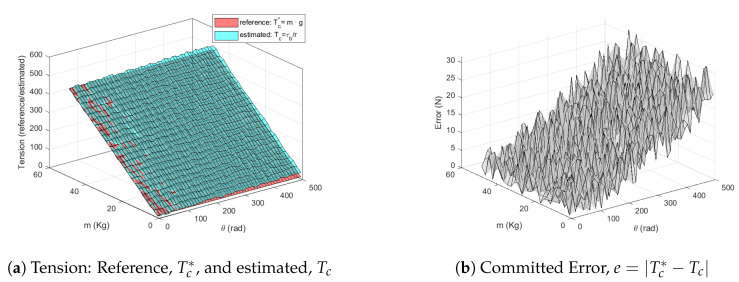
Tension estimation results.

Figure 10a compares the estimated cable tension, $T_{c}$, to the referenced one, $T_{c}^{*}$, for different mass, *m*, and different angular position, θ. Figure 10b represents the committed error, $e=|T_{c}^{*}-T_{c}|$. The results show that, in the tension estimation, the committed error slightly grows up when θ increases; nevertheless, the committed error is lower that 8% of the tension in the entire range of mass and angular displacement of the drum.

### 3.3. Actuator System Validation

The electromagnetic brake that is detailed in Section 2.4.2 allows for exerting an opposition torque at the drum axis, $\tau_{b}$, which originates an opposition force $F_{b}$ to the swimmer customized opposition force, $F_{b}\left(t\right)$, being commanded by a reference voltage. In order to guarantee that the reference opposition force fits to the real opposition force, an analogous procedure to the one that was defined to the torquemeter validation has been carried out.

**Figure 11:**
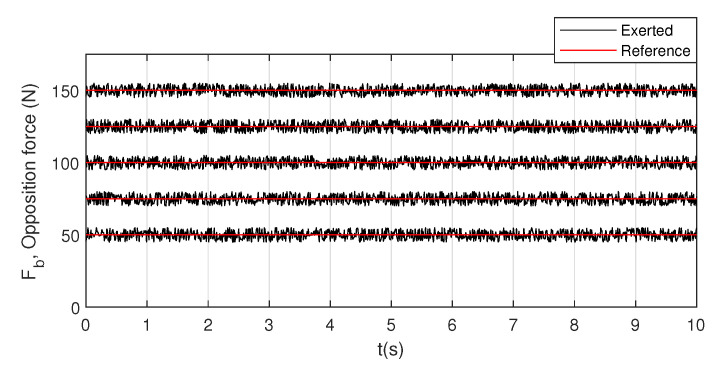
Examples of five experiments of opposition force validation.

For illustrative purpose, Figure 11 represents several experiments for different levels of opposition force: 50, 75, 100, 125, and 150 N.

### 3.4. Experimental Protocol

The experimental tests have been carried out with four males swimmers (age: 22.67±1.15 years; body mass 71.0±5.46 kg; height: 171.8±4.95 cm; training hours: >10 h per week (data are shown in Mean ± SD)). Each swimmer performed the trials on one stroke. Swimmers race at the national and regional championship level and they had minimum of 5 years’ experience at the national and regional competitive level.

All of the swimmers were familiar with strength training, but none of them had experience with resistance training using a device, such as SwimOne. The experimental procedures were fully explained to the participants, who were informed of the risks that are involved in the experiments and provided written consent before they participated in the study. The study was approved by the institutional ethics committee and it was in accordance with the Declaration of Helsinki [21].

A pilot study was carried out one week before the study in the same pool, where the tests were later performed, determining the position of the device, the data processing, and the procedure carried out on the testing day.

The participants visited the swimming pool in a non-fatigued state (non-intense exercise in the previous 48 h and avoid strength in the previous 72 h) and without taking any stimulating substance 48 hours before.

Each swimmer performed two trials without additional resistance in order to minimize any potential learning effects; previously, each one performed a 400m standardized warm-up consisting of 200 m easy front crawl, 100 m (12.5 fast and 12.5 easy) and 100m (25 kick and 25 swim). Each participant performed the tests at the same time of day and in the same swimming pool with a constant conditions ($27.5^{\circ }$ temperature, 55−60 HR, and density 0.997 g/cm^3^).

The swimmers performed eight sprints of 15 meters with a progression in the opposition force, $F_{b}$, which was produced by the device. The progression was established in an increment of 5 Nm in each sprint (0, 5, 10, 15, 20, 25, 30, 35).

The sprints were realized with 5 min. rest period before the next attempts in the following order: butterfly, backstroke, breaststroke, and front crawl. The subjects were wearing a belt attached to a steel cable. At the signal, the swimmer adopts a horizontal extended position next to the edge of the pool with the legs extended. At the next signal, the swimmer swims at maximum velocity for 15 m after pushing off from the wall. Each sprint was performed until reach 15 m (a mark was positioned in the surface), except in the last attempts, in which the swimmers cannot reach the 15 meters (due to the great resistance that is generated by the device); in these cases, the swimmers were stopped when the duration of the trial exceeds 20 s.

### 3.5. Results

**Figure 12:**
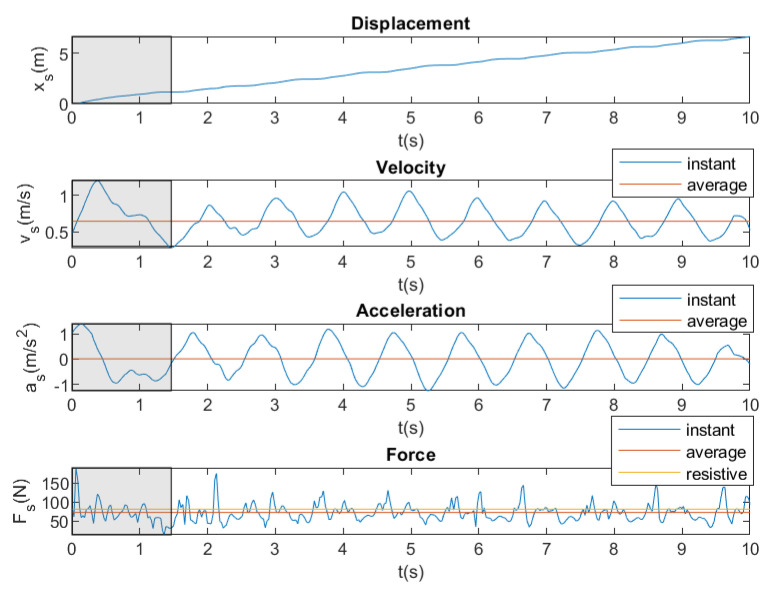
Instantaneous and average displacement, velocity, acceleration and force. Breaststroke. Brake force = 81.58 N.

Figure 12 shows an example of monitoring instantaneous displacement, velocity, acceleration, and force for breaststroke with a brake force of 81.58 N. Additionally, average velocity, acceleration, and force are monitored in Figure 12. In all of the figures, there is an area that is marked in gray that corresponds to the initial time when the swimmer is starting by kicking the wall; therefore, it is not considered for the calculation of the average values.

**Figure 13:**
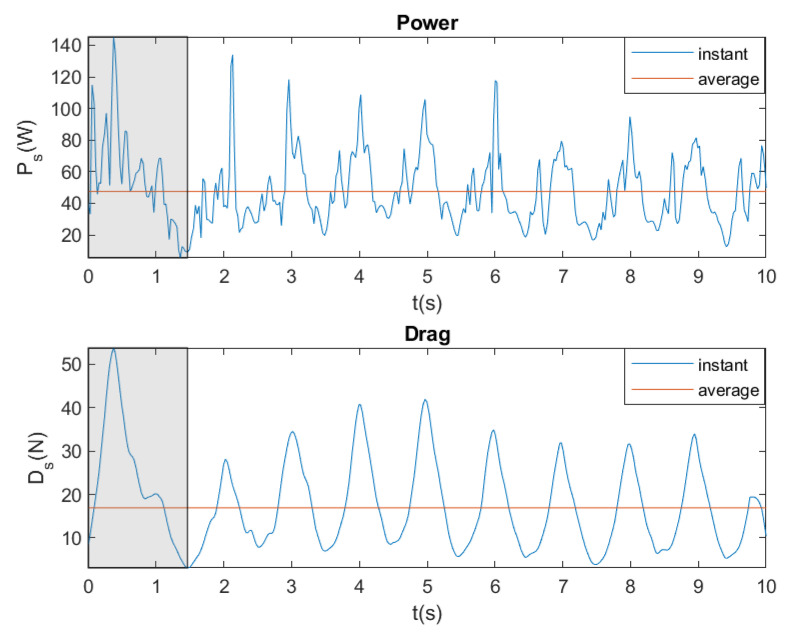
Instantaneous and average power and drag. Breaststroke. Brake force = 81.58 N.

Figure 13 shows an example of the results that were obtained monitoring instantaneous power and drag calculation. Additionally, average power and drag are shown in Figure 13. In both Figure 12 and Figure 13, the strokes performed by the swimmer can be clearly distinguished.

**Figure 14:**
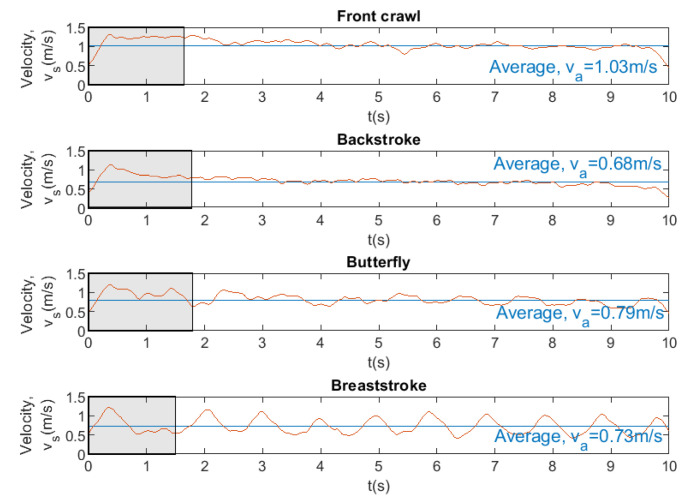
Instantaneous and average velocity for four swimming strokes. Brake force = 68.41 N.

**Figure 15:**
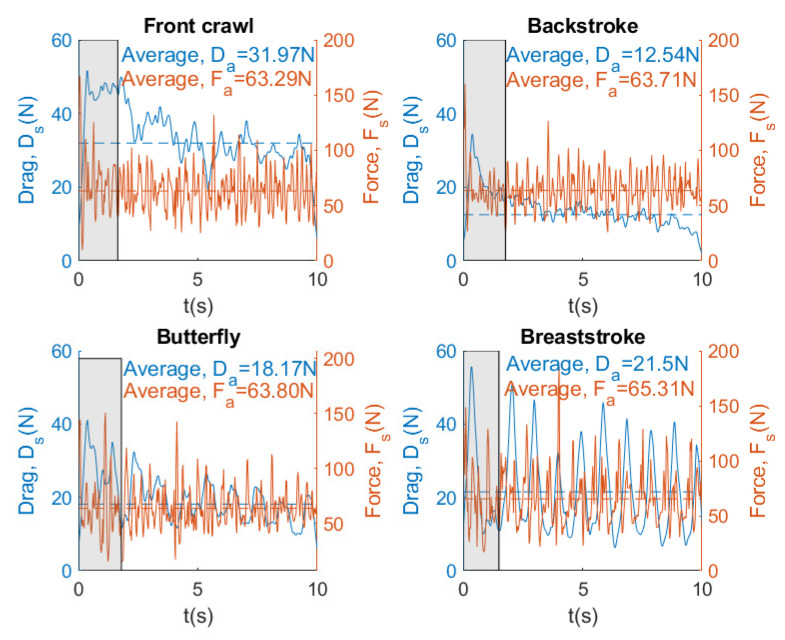
Instantaneous and average force and drag for four swimming strokes. Brake force = 68.41 N.

**Figure 16:**
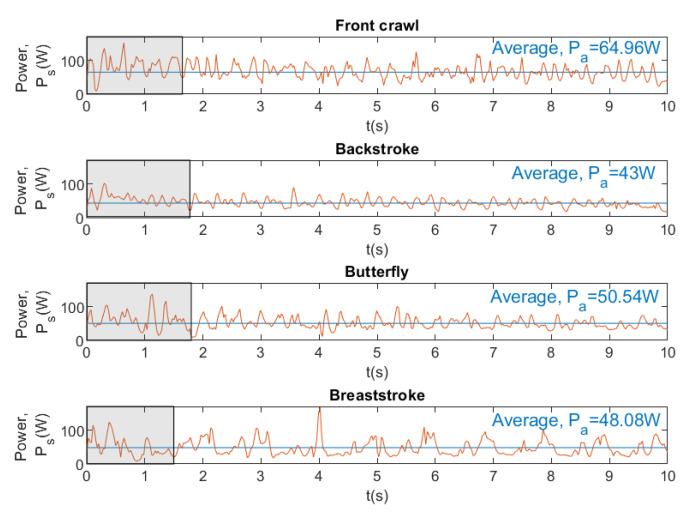
Instantaneous and average power for four swimming strokes. Brake force = 68.41 N.

Figure 14, Figure 15 and Figure 16 show the instantaneous and average velocity, force, drag, and power generated by the same swimmer for four swimming strokes with a brake force of 68.41 N.

From Figure 14, it can be observed that the fastest swimming stroke is the front crawl with an average velocity of 1.03 m/s, while the slowest swimming stroke is the backstroke with an average velocity of 0.68 m/s. In Figure 15, the results show that the higher velocity the greater the resistance that water exerts on the body. On the other hand, it can also be seen that the force that is exerted by swimmer in the four swimming strokes is very similar (64.03 N ±0.88). Figure 16 shows that the higher velocity the greater the power exerted by swimmer, as occurs with drag. Finally, it can be concluded that SwimOne allows for evaluating the performance of the swimmer in the different swimming strokes and at the same brake force.

**Figure 17:**
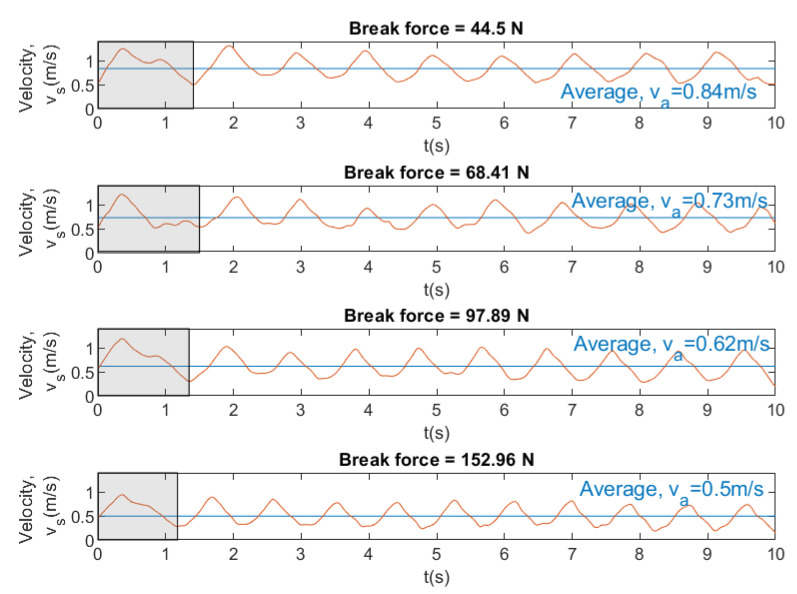
Instantaneous and average velocity for swimming stroke breaststroke at different brake forces.

**Figure 18:**
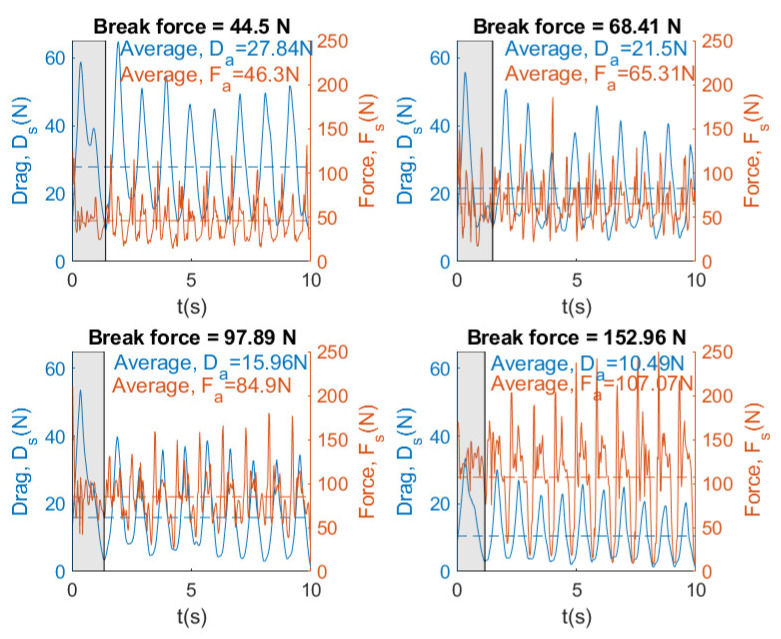
Instantaneous and average force and drag for swimming stroke breaststroke at different brake forces.

**Figure 19:**
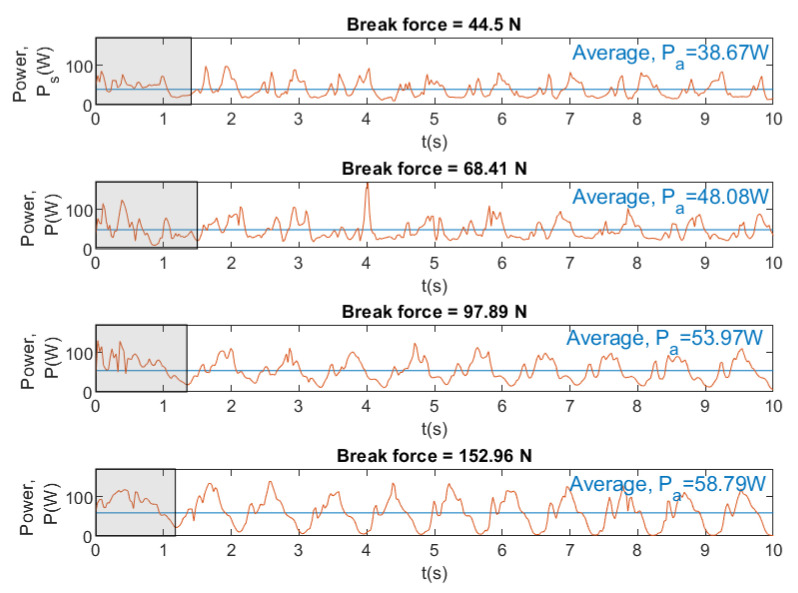
Instantaneous and average power for swimming stroke breaststroke at different brake forces.

Figure 17, Figure 18 and Figure 19 show instantaneous and average velocity, force, drag, and power for breaststroke swimming stroke by the same swimmer at different brake forces (44.5, 68.41, 97.89 and 152.96 N).

Figure 17 and Figure 18 show that, when the brake force increases, the velocity and the drag of the swimmer decreases. In Figure 18 and Figure 19, the results show that an increase in brake force yields an increase in the force and power. This makes sense, because the swimmer has to overcome a greater force of the brake and, therefore, has to exert a greater power. Finally, it can be concluded that SwimOne allows for the performance of the swimmer to be evaluated for single swimming stroke and at different brake forces.

**Figure 20:**
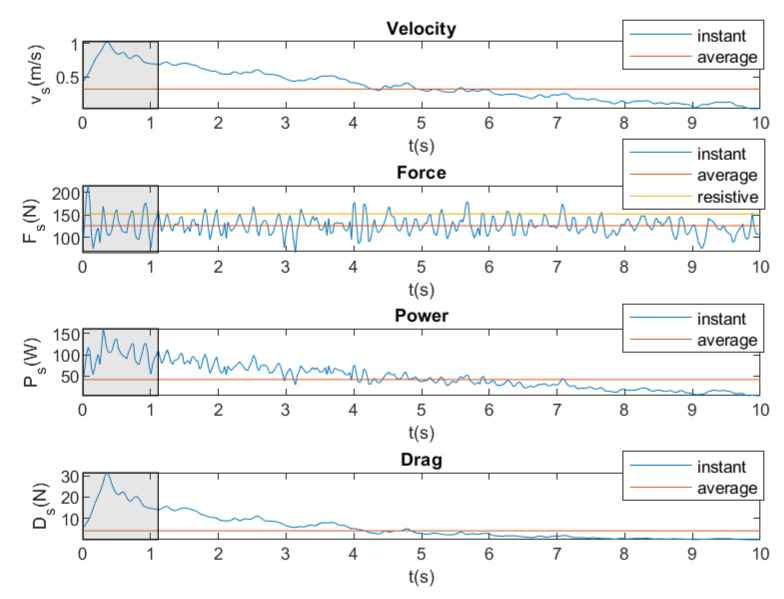
Instantaneous and average velocity, force, power, and drag. Front crawl. Brake force = 152.96 N.

Figure 20 shows the instantaneous and average velocity, force, power, and drag for front crawl swimming stroke by the same swimmer with a brake force of 152.96 N.

In Figure 20, the results show that brake force ($F_{b}$) is greater than the force that is exerted by the swimmer ($F_{s}$). Therefore, the velocity, the drag, and the power of the swimmer will decrease until the swimmer does not generate any displacement, since it does not overcome the opposing force that is exerted by the brake. Finally, it can be concluded that SwimOne allows for determining the maximum opposing force that the swimmer is able to overcome.

**Figure 21:**
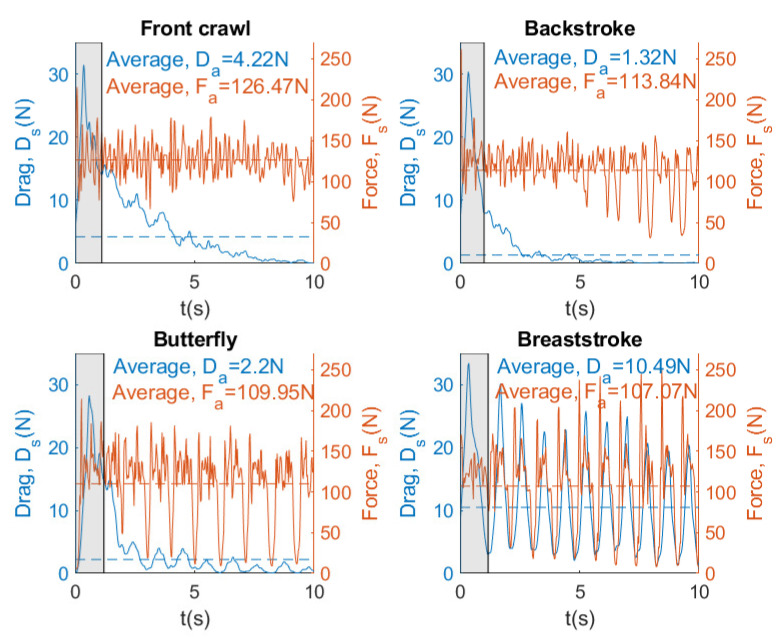
Instantaneous and average force and drag for four swimming strokes. Brake force = 152.96 N.

Figure 21 shows instantaneous and average force and drag for four swimming strokes by the same swimmer with a brake force of 152.96 N.

From Figure 21, it can be observed that the resistance of the swimmer decreases in the front crawl, backstroke, and butterfly swimming strokes. This is because the swimmer cannot overcome the force exerted by the brake and slows down until no displacement is observed. In this case, it can also be seen that the force exerted by swimmer in the four swimming strokes is very similar (114.49 N ±8.38). Finally, it can be concluded that SwimOne allows for determining in which of the swimming strokes the swimmer has to improve his performance.

## 4. Results Discussion

The aim of the current study is to validate the SwimOne device for evaluating propulsive forces in swimming with additional resistance or free swimming without resistance. Propulsive force was recording as average propulsive force ($F_{a}$) in absolute data [4,22,23]. $F_{a}$ is the more accurate variable for assessing force production in the tethered and semi-tethered swimming test [17]. In fact, $F_{a}$ showed a strong relationship with the velocity in 50 m, 100 m, and 200 m freestyle events [4,19].

Our results showed that $F_{a}$ value (126 N) was achieved in freestyle in the last attempt with 153 N of break force, which can be considered as a tethered swim test due to the registered velocity (0.3 m/s). In [19] similar values of $F_{a}$ were reported (133.2±16.8 N), carrying out tethered trials using front crawl technique. However, it may be observed several differences between strokes. Front crawl presents higher $F_{a}$ values (126.17 N), followed by backstroke (113.84 N, 9.8% less than crawl), butterfly (110.93 N, 13.5% less than crawl), and breaststroke (107.07 N, 14.1% less than crawl) (see Figure 21). Butterfly and breaststroke obtained lower values of $F_{a}$ as a result of the simultaneous actions of both arms and legs and, consequently, leading to a higher intracycle velocity variation [24]. One additional reason for these low values of $F_{a}$ could be the fact that the swimmers are not breaststroke specialist. These intracycle variations are accentuated as the brake force also increased in force and power exerted by a swimmer, showing a greater amplitude between the intracycle peaks, especially in breaststroke. However, the swimming pattern remained constant (Figure 17, Figure 18 and Figure 19). Therefore, front crawl and backstroke have been the strokes with lower intracycle velocity variation (Figure 14).

Despite the differences in $F_{a}$ between strokes with the maximal load (153 N), in a sub-maximal load (68 N) these differences only present a slight variation between strokes. Thus, the velocity variation between strokes during sub-maximal load trials would be associated with differences in drag instead of $F_{a}$ (Figure 15).

The intracyclic velocity variation between strokes has a direct influence on the power output that is generated by the swimmers. According to our results, in front crawl, it can be observed that MSP (maximum swim power) (66.74 W) was obtained with 68N of brake that represents 44.52% of the maximal load (it is usually expressed in this way for dry-land power) [25]. It is in line with the results of several studies, as the research in [22] at which MSP (66.48 W) was obtained with a 47.07% of the maximal load. Work [26] also indicated that MSP is found at 44.52% of the maximal load showing MSP values around 57 W.

It should also be highlighted that MSP was quite different between strokes and also in the point in which it was achieved. In this sense, front crawl showed the higher values (66.74 W), following by breaststroke (58,79 W), butterfly (51.13 W), and backstroke (43.29 W) (Figure 16). These differences can be explained due to the variation of velocity between strokes. Furthermore, MSP was achieved with different resistive forces between strokes; in breaststroke, it was achieved at 100% of maximal load; whereas, in backstroke, front crawl and butterfly it was achieved at 44.52% of maximal load; however, the vast majority of the semi-tethered studies were performed with front crawl, leaving a lack of analysis regarding to other swimming strokes [7]. Nevertheless, in [27] the power delivered to an external weight in the four swimming techniques was measured, obtaining higher correlations between MSP and performance for breaststroke (r=−0.90), followed by butterfly (r=−0.89), backstroke (r=−0.84), and freestyle (r=−0.80).

## 5. Conclusions

This paper presents a new device for estimating instantaneous propulsive force and the power of swimmers. The conceptual idea is presented by describing the differential equation of the swimmer dynamics. The device is presented as a first prototype, describing the technical requirements and consequent sensor and actuator systems.

The protocol for estimating the instantaneous power of the swimmer by means of the acquisition of angular position of the drum and the cable tension is detailed.

The device allows for the evaluation of force and power production, independently of the technique performed, which is a very useful tool for monitoring and evaluating the training loads and, as a consequence, the performance progression in power, force, velocity through the season. The device can be used as an additional method to develop swim power, as well as in dry-land exercises in which, through a linear encoder, it possible to set force-velocity-power curves and establish the optimal loads and velocities for improving the specific power [28,29].

The device allows for determining the optimal zones for improving the specific power in swimming in a real situation, in which the swimmer performs the power training with the same arms and legs trajectories and body position that are carried out on the swimming strokes.

In conclusion, SwimOne was shown to be a valid device for measuring instantaneous and average force and power with different loads in swimming [30,31]. The results of power and force are in line with previous studies that used tethered and semi-tethered as a method for quantifying propulsive force in swimming. The device also allows for quantifying the improvements obtained with the training plan. It is a very useful information for coaches and swimmers to adapt the training loads and training cycles. Moreover, the device could be used as a training tool for improving the specific strength and power in a very similar situation to swimming.
